# *MIR137* is the key gene mediator of the syndromic obesity phenotype of patients with 1p21.3 microdeletions

**DOI:** 10.1186/s13039-016-0289-x

**Published:** 2016-11-03

**Authors:** Arianna Tucci, Claudia Ciaccio, Giulietta Scuvera, Susanna Esposito, Donatella Milani

**Affiliations:** Pediatric Highly Intensive Care Unit, Department of Pathophysiology and Transplantation, Università degli Studi di Milano, Fondazione IRCCS Ca’ Granda Ospedale Maggiore Policlinico, Via Commenda 9, 20122 Milano, Italy

**Keywords:** Obesity, Array-CGH, Genetics, *MIR137*, 1p21.3, Intellectual disability, Autism spectrum disorder

## Abstract

**Background:**

Deletions in the long arm of chromosome 1 have been described in patients with a phenotype consisting primarily of obesity, intellectual disability and autism-spectrum disorder. The minimal region of overlap comprises two genes: *DPYD* and *MIR137*.

**Case presentation:**

We describe a 10-year-old boy with syndromic obesity who carries a novel 1p21.3 deletion overlapping the critical region with the *MIR137* gene only.

**Conclusions:**

This study suggests that *MIR137* is the mediator of the obesity phenotype of patients carrying 1p21.3 microdeletions.

## Background

Obesity is a complex disease that results from the interactions of a variety of hereditary and environmental factors. Heritability for obesity is high (typically 0.4 to 0.7) [1], comparable to that of other complex, polygenic diseases such as autism [2]. In most individuals, the genetic basis for obesity is complex and likely to involve the interaction of multiple genes and gene-by-environment interactions. However, there are rare examples of monogenic causes for obesity that provide insights to pathways that may account for more common causes of obesity and indicate targets for therapeutic intervention. These disorders include obesity syndromes such as Prader Willi syndrome and Bardet–Biedl syndrome and microdeletion/duplication syndromes such as 1p36 and 17p11.2, in which affected patients are usually identified by additional phenotypes, such as intellectual disability (ID), dysmorphic features or other developmental abnormalities. Gaining insight into genetic causes through the study of obesity-related disorders has provided a more comprehensive picture of the mechanisms that control food intake and energy balance related to the development of obesity (e.g., *SIM1*).

Deletions in chromosome 1p21.3 have been reported in a total of 12 individuals presenting with a variable combination of ID, autism spectrum disorders (ASD) and obesity [3–6]. The shortest region of overlap (SRO) has been restricted to two genes: dihydropyrimidine dehydrogenase (*DPYD*) and microRNA 137 (*MIR137*). The *DPYD* encodes for the rate-limiting enzyme in the catabolism of the pyrimidine bases, and mutations in this gene cause DPYD deficiency (OMIM ♯274270), an autosomal recessive disease of variable severity typically characterized by the presence of childhood onset neurological problems, such as developmental delay and convulsions. *MIR137* encodes for miR-137, a microRNA (miRNA) molecule involved in the post-transcriptional regulation of genes [7].

In this study, we report an additional case carrying a 1p21.3 microdeletion that presented with ID, ASD, obesity and additional clinical features. We propose the *MIR137* gene as the mediator of the obesity phenotype that presents in patients carrying chromosome 1p21.3 microdeletions.

## Case presentation

The proband is a ten-year-old male and the first-born child of healthy non-consanguineous parents. The family history was unremarkable for neurological disorders, behavioral problems, and congenital anomalies.

He was born at 39 weeks of gestation via elective caesarian section. His birth weight was 2800 g (10-25th centile) and his length, occipital frontal circumference and APGAR scores were not reported but were said to be normal. At birth, no medical problems were recorded. Postnatally, his growth was normal; global psychomotor delay was noted: the boy took his first steps at the age of 20 months and said his first words at the age of 36 months. In infancy, he had several episodes of febrile convulsions, which spontaneously resolved at the age of 3 years. During a routine heart examination, a heart murmur was noticed, which prompted further tests. An echocardiogram showed a bicuspid aortic valve, with mild stenosis of the aortic root.

At the age of 9, he attended the fifth year of primary school due to speech disorder and anxiety. Neuropsychiatric assessment revealed anxiety, normal non verbal cognitive function, specific language impairment and fine motor skills disturbances, requiring weekly psychomotricity sessions. His growth parameters were as follows: weight was 39.3 kg (75th centile), height 147.2 cm (97th centile), body mass index (BMI) was 18.13 kg/m^2^. At the age of 10, the boy weighted 48 kg (>97th centile), height was 154 cm (>97th centile), head circumference was 58 cm (>97th centile) and BMI was 20.6. Pubic hair appearance, growth spurt and weight gain were noted. The boy was hospitalized, and further tests were performed, confirming precocious puberty of central origin: a radiograph of the hand and wrist, which showed advanced bone age (bone age of 13 years, biological age 9 years and 5 months); renal echography, adrenal glands and tests, which were normal, and the LHRH test, which showed an LH response. The boy started agonist LHRH therapy. Brain magnetic resonance imaging (MRI) showed increased cerebrospinal fluid (CSF) spaces posteriorly to the cerebellum and a moderate focal increase of CSF in the anterior temporal horns and in the lamina of the quadrigeminal cistern. The pituitary gland was normal.

Chromosome analysis by array-CGH (a-CGH) revealed a 5.2-Mb deletion on chromosome 1p21.3p21.1 (chromosome 1:98,456,293-103,682,084 – hg19). a-CGH was performed using a Cytochip ISCA 180 K Oligo platform (BlueGnome Ltd.). a-CGH analysis of the parents revealed the de novo origin of the deletion.

## Conclusions

**Fig. 1:**
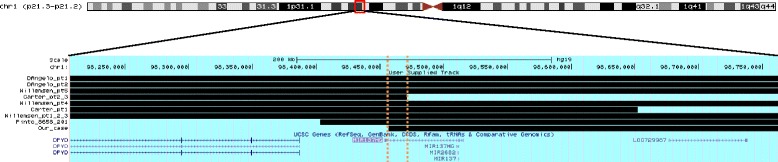
Chromosome 1p21.3 microdeletions described to date. Minimal region of overlap highlighted by the *dashed orange lines*. The figure was drawn according to the UCSC Genome Browser on Human Feb. 2009 (GRCh37/hg19) assembly

Deletions at 1p21.3 have been recognized as a cause of childhood obesity syndrome. Twelve patients carrying 1p21.3 deletions have been described in the literature to date [3–6], presenting with mild to moderate ID (100 %), obesity or a tendency to obesity, with a weight 3 or more standard deviations above the mean (92 %), ASD (92 %) and mild dysmorphic features (67 %) (Fig. 1 and Table 1). The minimal overlapping region was restricted to a chromosomal segment of 1.22 Mb, which includes the *DPYD* and *MIR137* genes. *DPYD* was proposed as the gene contributing to the phenotype [3, 5], based on the assumption that its haploinsufficiency would contribute to the development of a neuropsychiatric phenotype, which also leads to obesity. However, the case herein presented and the patient recently reported by Pinto et al. [6] carried a deletion overlapping in the critical region only at the *MIR137*, which suggests that this is the gene underlying the phenotype of patients carrying deletions at 1p21.3.

**Table 1:** Clinical features of patients carrying overlapping 1p21.3 deletions

	Carter pt 1	Carter pt 2, pt 3	Willemsen pt 1, pt2, pt 3	Willemsen pt 4	Willemsen pt 5	D’Angelo pt 1	D’Angelo pt 2	Pinto pt 8658_201	Our case
Chromosome Position on chr 1 (hg19)	97,559,579–98,652,079	96,969,562–98,471,225	97,727,412–99,477,412	97,547,412–98,957,412	96,497,412–98,947,412	93,919,217–99,846,176	95,696,444–107,755,879	98,403,034–101,151,364	98,456,293–103,682,084
Age	13 years 9 months	7 years, 5 years	42 years, 38 years, NA	33 years	18 years	15 years	8 years 8 months	NA	10 years
Gender	M	M, F	M, M, F	M	F	F	F	F	
Intellectual disability	Severe language delay	Severe language delay	Borderline, Mild-moderate	Mild	Moderate	Language delay	Severe language delay	Severe language delay	Mild
Autism spectrum disorder	+	+	+	+	+	NA	+	+	+
Weight	NA	> > 97^th^ centile 50th centile,	90^th^, >98^th^ centile	98^th^ centile	>98^th^ centile	>95^th^ centile	>95^th^ centile	Overweight	Overweight
Dysmorphic features	NA	+	+	+	+	+	-	NA	-
Ocular problems	NA	NA	Myopia, astigmatism	Myopia, astigmatism	/	Myopia	/	NA	Hypermetropy
Precocious puberty	NA	NA	-	-	-	-	+	NA	+
Others	/	Small joint hypermobility, macrocephaly	/	/	/	/	/	High pain intolerance	Bicuspic aortic valve

miRNAs are noncoding 21–23 nucleotide small RNAs that are highly conserved and involved in several biological processes, such as cell proliferation, apoptosis, cell differentiation and morphogenesis [8]. They regulate posttranscriptional processes via sequence-specific interactions with the untranslated regions (UTRs) of cognate messenger RNA targets [7, 9]. Each individual miRNA can regulate many mRNAs, and conversely, each mRNA can be targeted by a number of miRNAs. The involvement of miR-137 in obesity, ID and ASD could be explained at two different levels. First, miR-137 regulates a multitude of genes in the central nervous system (CNS) [10]. Specifically, miR-137 has been shown to be involved in modulating neurogenesis, with a dose-dependent effect [11]. Moreover, the miR137 targets Ezh2, a H3-K27 methyltransferase involved in the regulation of neuroprogenitor cell maintenance and differentiation [12]. Notably, mutations in the *EZH2* gene cause Weaver syndrome, another syndromic overgrowth disorder characterized by ID, dysmorphic features and obesity [13]. Although the role of neuronal noncoding RNAs in the energy control of the body is not fully understood, recently, the hypothalamic miR-103 has been shown to protect against hyperphagic obesity in mice [14]. Therefore, the role of miR-137 in the control of food intake or energy accumulation and expenditure in the CNS can be speculated.

At a peripheral level, recent studies have shown that miRNAs are dysregulated in obese adipose tissue [15]. Furthermore, during adipogenesis, miRNAs regulate fat cell development by accelerating or inhibiting adipocyte differentiation. In addition, miRNAs may regulate the commitment to the adipogenic lineage in multipotent stem cells and therefore govern the numbers of fat cells [16].

Ninety-two percent of the cases carrying 1p21.3 deletion present with ASD. miR-137 is a brain expressed miRNA [17, 18] that regulates several protein coding genes that are important for brain function, and involved in the pathogenesis of neuropsychiatric disorders including schizophrenia [17, 19]. Functional studies indicate that miR-137 is involved in controlling neuronal differentiation and maturation [20, 21] as well as synapse development [22], all of which have been implicated on the pathogenesis of autism [20, 23]. Of note, lymphoblastoid cell lines from patients carrying 1p21.3 deletion involving the miR-137 gene have reduced levels of miR-137 [4]. miR-137 is highly expressed in the hippocampus, occipital cortex, and frontal cortex in human post-mortem tissue, as well as in the synaptosomal fractions in mouse brain preparations, providing further evidence that miR-137 plays a role in synapse formation during brain development [4].

Several studies have implicated miRNA in autism. A number of miRNAs have shown differing expression levels in samples from autistic individuals compared to matched controls [24, 25], suggesting that dysregulation of miRNAs could be related to autism. Furthermore, miR-137 has been recently implicated in ASD via a genome-wide meta-analysis that considered single nucleotide polymorphism data across five psychiatric disorders (ASD, attention deficit-hyperactivity disorder, bipolar disorder, major depressive disorder, and schizophrenia), and found an association between mir-137 and both schizophrenia and ASD [26].

Lastly, Devanna and Vernes provided support for miR-137 as an autism candidate by showing that the RORa gene, a novel autism candidate gene, is regulated directly by miR-137 through targeting its 3 UTR in a site-specific manner [27].

It is therefore likely that the core clinical features of obesity and ASD in patients carrying 1p21.3 deletion are both mediated by miR-137.

The case herein described presents with clinical features not previously described in the 1p21.3 microdeletion syndrome, such as precocious puberty and bicuspid aortic valve, with mild stenosis of the aortic root. The boy carries a 1p21.3 deletion, which extends 5 Mb proximally to the SRO and includes a number of genes. *PALMD* and *S1PR1* encode for proteins that are expressed during normal embryonic heart development and morphogenesis. Specifically, PALMD has been associated with aortic root size [28], while S1PR1 has an important role in the regulation of sprouting angiogenesis and vascular maturation. It inhibits sprouting angiogenesis to prevent excessive sprouting during blood vessel development [29]. With regard to precocious puberty, we found two DECIPHER patients that carried deletions partially overlapping with our case and precocious puberty: case 260349, which presented with precocious puberty and specific learning disability and carrying a deletion at chr1:97,000,749–99,813,665; case 254871 presented with ASD, ID, precocious puberty and spotty hyperpigmentation, carrying the deletion at chr1:97019762–102447536. The SRO among these microdeletions includes the SNX7, PLPPR4 and PLPPR5 genes. Both PLPPR4 and PLPPR5 encode for a lipid phosphate phosphatase enzyme specifically expressed in neurons and located in the membranes of outgrowing axons, important for axonal outgrowth during development and regenerative sprouting [30, 31].

In summary, with this study, we present a case carrying a novel 1p31.3 microdeletion, and we propose that the miRNA miR137 is the mediator of the obesity, ASD and ID phenotype. Further studies are warranted to elucidate the molecular mechanisms by which miR-137 mediates the phenotype, both in the CNS and the adipose tissue.

## Abbreviations

ASD: Autism-spectrum disorders
BMI: Body mass index
CNS: Central nervous system
CSF: Cerebrospinal fluid
ID: Intellectual disability
MRI: Magnetic resonance imaging
SRO: Shortest region of overlap
UTRs: Untraslated regions
